# Systemic consequences of abnormal cholesterol handling: Interdependent pathways of inflammation and dyslipidemia

**DOI:** 10.3389/fimmu.2022.972140

**Published:** 2022-08-26

**Authors:** Ross O’Hagan, Alex R. Berg, Christin G. Hong, Philip M. Parel, Nehal N. Mehta, Heather L. Teague

**Affiliations:** Section of Inflammation and Cardiometabolic Diseases, National Heart, Lung, and Blood Institute, National Institutes of Health, Bethesda, MD, United States

**Keywords:** cholesterol handling, dyslipidemia, inflammation, immunometabolism, psoriasis

## Abstract

Metabolic conditions such as obesity and associated comorbidities are increasing in prevalence worldwide. In chronically inflamed pathologies, metabolic conditions are linked to early onset cardiovascular disease, which remains the leading cause of death despite decades of research. In recent years, studies focused on the interdependent pathways connecting metabolism and the immune response have highlighted that dysregulated cholesterol trafficking instigates an overactive, systemic inflammatory response, thereby perpetuating early development of cardiovascular disease. In this review, we will discuss the overlapping pathways connecting cholesterol trafficking with innate immunity and present evidence that cholesterol accumulation in the bone marrow may drive systemic inflammation in chronically inflamed pathologies. Lastly, we will review the current therapeutic strategies that target both inflammation and cholesterol transport, and how biologic therapy restores lipoprotein function and mitigates the immune response.

## Introduction

Chronic inflammatory conditions have significantly higher rates of cardiometabolic disorders associated with abnormal cholesterol levels and poor cholesterol trafficking. In diseases of cholesterol accumulation, it is becoming evident that cholesterol handling plays a consequential role in driving systemic inflammation and promoting cardiovascular disease (1). Moreover, in chronic inflammatory conditions such as psoriasis, cholesterol accumulation may be the culprit in rampant inflammation, insulin resistance and a heightened risk of cardiovascular disease (2, 3). In this review, we summarize the current literature on the interdependent relationships between abnormal cholesterol handling and systemic inflammation, and their connection to cardiovascular disease.

## Abnormal cholesterol transport

It is well known that unfavorable cholesterol profiles contribute to atherosclerosis, leading to life-threatening complications such as stroke, and confers a heightened risk of cardiovascular events and mortality (4–6). Cholesterol accumulation is often described in reference to the vasculature, however, a reduction in systemic cholesterol transport would result in accumulation throughout the periphery and may overload the intracellular compartments. Evidence of this may be found in studies of dementia that show cholesterol accumulation at mid-age is associated with an elevated risk of cognitive decline (7). Further, Niemann-Pick disease, a condition with impaired intracellular cholesterol transport, displays both cognitive and respiratory impairment, among other inflammatory ailments (8). Removal of excess cholesterol from peripheral tissues for excretion is the primary role of high-density lipoprotein (HDL), measured as cholesterol efflux capacity (CEC). Over the last decade, clinical trials have focused on increasing HDL quantity in an effort to reduce cardiovascular events. However, minimal improvements were observed and in some cases adverse events were reported (9). HDL function, which can be measured as CEC (10), is now being investigated as both a diagnostic and therapeutic target, to improve cardiovascular event prediction and improve outcomes.

## Cholesterol efflux capacity and cardiovascular events

Over the last decade, epidemiological studies have reported elevated risk of cardiovascular disease and events with impaired CEC, suggesting CEC may contribute to atherosclerosis initiation and progression (10, 11). CEC has an inverse association with carotid intima-media thickness and the likelihood of angiographic coronary artery disease, independent of HDL cholesterol levels (10). In the JUPITER (Justification for the Use of Statins in Prevention: An Intervention Trial Evaluating Rosuvastatin) trial, CEC independently correlated with cardiovascular events in a cohort of mostly white male participants on intensive statin therapy (12). The PREVEND (Prevention of Renal and Vascular End-stage Disease) cohort determined that CEC predicted cardiovascular events in the general population, independent of apolipoprotein-A1 (ApoA-1) and HDL (13). Moreover, the multiethnic Dallas Heart Study showed when patients without CVD were stratified by CEC quartiles in fully adjusted models, the highest quartile of CEC had a 67% reduction in cardiovascular risk compared to the lowest quartile (1). Further, the study found that while black men and women had significantly higher rates of CVD, no difference in CEC was detected beyond adjustment (14). In the event of a myocardial infarction (MI), CEC may be protective; with increased serum CEC associated with a lower rate of mortality after an MI (15). Interestingly, one study found that heightened CEC with concurrent ApoB depletion increased the risk of MI and stroke over time, suggesting cholesterol elimination below a certain threshold may be detrimental to vascular health (16). Curiously, while women are at a lower risk of CVD, and CEC levels are enhanced by estradiol levels, there is no significant difference in CEC between sexes (17). Taken together, these findings highlight the critical role of CEC in vascular health and suggest that targeting CEC for therapeutic development may prove efficacious in the reduction of cardiovascular events.

## Interdependent pathways between cholesterol and immunity

Cholesterol uptake within immune cells and peripheral tissues occurs through the lysosomal compartments by receptor-mediated endocytosis. Cholesterol is hydrolyzed by lipoprotein lipase in the lysosome into free cholesterol and a fatty acid (18). Cholesterol is then shuttled to either the plasma membrane to maintain membrane integrity and homeostasis, or to the endoplasmic reticulum for conversion into hormones, bile salts or efflux out of the cell. Nuclear factor erythroid 2 related factor-1 (NRF1) is a receptor in the endoplasmic reticulum that functions as a cholesterol sensor to maintain intracellular homeostasis (19). In the liver, high intracellular cholesterol load prompts NRF1 to upregulate ATP-binding cassette subfamily A member 1 (ABCA1) expression for cholesterol export out of the cell, requiring optimal HDL function to receive cholesterol. However, failure to remove excess cholesterol leads to intracellular accumulation and the inflammatory response is primed (19). In macrophages, accumulation of cholesterol is tightly linked to atherosclerotic plaque formation in the coronary arteries (20). Removal of cholesterol through efflux to ApoA-1 on HDL is the primary method of cholesterol displacement and this process occurs through ABCA1 or ABCG1 on macrophages that directly interacts with ApoA-1. The interaction of ABCA1 or ABCG1 with HDL is dependent upon HDL size; larger HDL molecules receive cholesterol through ABCG1 compared to ABCA1, which typically delivers cholesterol to smaller HDL molecules (21). Cholesterol efflux through ABCA1 and ABCG1 modulates downstream immunological signaling. In macrophages specifically, efflux through ABCA1 and ABCG1 downregulates toll-like receptor 2, toll-like receptor 3, and toll-like receptor 4 expression, which reduces nuclear factor−κB (NF-kB) expression and thus decreases inflammasome activation (22). Murine studies show that macrophages lacking ABC transporters have heightened tumor necrosis factor-alpha (TNF-α) secretion (22) and excess formation of lipid rafts, further stabilizing inflammatory signaling (23). Further, the removal of excess cholesterol from ABC-deficient macrophages decreases their inflammatory response and reduces lipid-raft formation (22). These findings are not specific to macrophages; atherosclerotic mice lacking ABCA1 have increased circulating neutrophils and more neutrophil extracellular trap (NET)-formation, an acute pro-inflammatory release of nuclear DNA (24).

Cholesterol crystals are an emerging area of research that may partially explain the observed relationships between cholesterol accumulation, immune cell activation and atherosclerosis. Cholesterol crystals are highly prevalent features in lipid-rich necrotic core, a high-risk coronary plaque feature found in late atherosclerosis (25). The formation of cholesterol crystals is thought to relate to a dysfunction in cholesterol transport, and the size and composition of these crystals are hypothesized to contribute to plaque rupture (25). Cholesterol crystals are known to activate the NOD-like receptor protein 3 (NLRP3) inflammasome in myeloid cells, perpetuating systemic inflammation (26) Cholesterol crystals expedite neutrophil extracellular trap (NET) formation, leading to T-helper (H)-17 cell recruitment (27). In addition, cholesterol crystals can accumulate in the lysosomes of monocytes and neutrophils, leading to lysosomal destabilization and downstream activation of NLRP3 inflammasomes (28). Taken together, this indicates that cholesterol accumulation causes a shift towards an inflammatory phenotype in myeloid cells, modulating the immune response.

**Figure 1:**
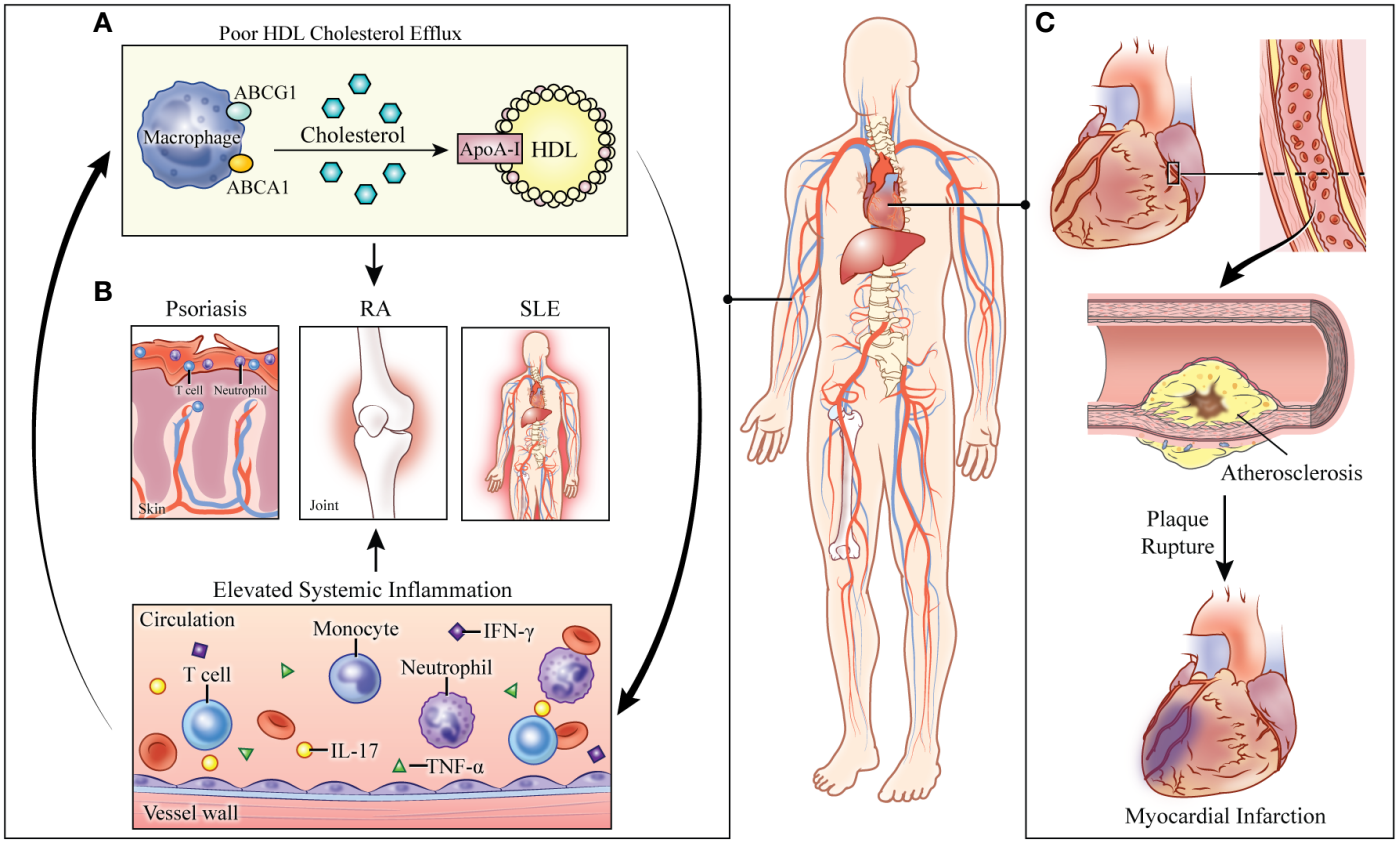
Consequences of abnormal cholesterol transport in systemically inflamed pathologies. **(A)** Defective cholesterol efflux capacity (CEC) perpetuates immune cell proliferation and activation. Elevated systemic inflammation further impairs cholesterol transport. **(B)** In the systemically inflamed diseases of psoriasis, rheumatoid arthritis (RA), and systemic lupus erythematosus (SLE), CEC is associated with disease severity. **(C)** The combined effects of systemic inflammation and defective cholesterol transport lead to increased risk of cardiovascular disease and myocardial infarction in these pathologies. HDL: high-density lipoprotein. ABCG1: ATP-binding cassette subfamily G member 1. ABCA1: ATP-binding cassette subfamily A member 1. ApoA-1: apolipoprotein A1. RA: rheumatoid arthritis. SLE: systemic lupus erythematosus. IL: interleukin. TNF-α: tumor necrosis factor-alpha. IFN- γ: interferon-gamma.

The observed *in vivo* relationships in preclinical models can be translated to clinical models. Patients with ABCA1 mutations have reduced CEC and HDL concomitant with increased amounts of TNF-α and interleukin (IL)-6. Positron emission tomography (PET) scans of these patients further revealed increased fluorodeoxyglucose (FDG)-measured inflammation of the aortic arch when compared to controls (29). Similarly, Tangier disease, an inherited disease caused by a loss of function mutation in the ABCA1 gene, associates with low HDL levels, decreased CEC and increased serum IL-1β and IL-18, pro-inflammatory cytokines released upon inflammasome activation (24). Indeed, these findings confirm the intricate relationship between dyslipidemia and systemic inflammation and suggest a role of CEC as a mediator between the two pathologies (**Figure 1**).

## Cholesterol and the bone marrow

Poor cholesterol trafficking and an overactive immune system are both prominent in cardiovascular disease and autoimmunity, with monocytes and neutrophils representing critical drivers (13). The linkage between dyslipidemia and innate immunity has been studied extensively in mice and translated to humans (30). In low-density lipoprotein (LDL)-receptor knockout mice, haploinsufficiency of ApoA-1 reduces HDL and ApoA-1, leading to the expansion of hemopoietic stem and progenitor cells (HSPC) into monocytes in the bone marrow (31). Further, deficiency in ABCA1 leads to cholesterol accumulation concomitant with hyperproliferation of monocytes and neutrophils, which is exacerbated by a high-fat diet (32). In humans, bone marrow derived HSPCs from patients with hypercholesteremia showed a migratory gene profile, indicating these cells were poised to exit the bone marrow (25), and their transcriptome was skewed towards monocyte proliferation (33). Familial hypercholesteremia (FH) patients have similar immune profiles; the circulating monocyte populations were continuously active in a state of trained immunity (34), which persisted despite treatment with lipid-lowering therapy that rectified high LDL cholesterol. This highlights that the residual inflammatory risk may be undeterred by reducing circulating cholesterol levels once the inflammatory response has been programmed. Additionally, LDL cholesterol from patients with FH associated with circulating monocyte populations and had an inverse relationship between monocytes and HDL (35). Moreover, in hyperlipidemic children, LDL cholesterol associated with monocytes (33). In pathologies of chronic inflammation with poor CEC, imaging studies showed that bone marrow activation assessed by PET-computed tomography (CT) associated with coronary artery disease, high-risk coronary plaque features, and monocyte and neutrophil populations (36). It is becoming evident that in humans, abnormal lipid profiles my lead to cholesterol accumulation in the bone marrow, and thereby reprogram HSPCs towards an active myeloid phenotype, similar to murine studies of atherosclerosis, warranting further investigations in the area (37).

## Impact of oxidized and modified lipids and on cellular cholesterol efflux and inflammation

Oxidation of HDL, LDL, and other lipid particles hamper cholesterol transport and induces inflammation. HDL can be oxidized by multiple biological mechanisms, with oxidation by neutrophil and macrophage-released myeloperoxidase (MPO) being the most understood (38). MPO-driven oxidization alters numerous amino acids within ApoA-1, compromising ApoA-1 function which leads to cholesterol accumulation in macrophages (39). ApoA-1 oxidized by nitration reduces the activation of lecithin: cholesterol acyltransferase (LCAT), hampering early steps in reverse cholesterol transport (40). Moreover, NF-kB activation is induced (41). Furthermore, the atherogenic effects downstream of ApoA-1 oxidation are associated with cardiovascular events (42). Like oxidized HDL, oxidized LDL (ox-LDL) is inflammatory and disrupts cholesterol transport. Once phagocytized by macrophages, ox-LDL contributes to cholesterol crystal formation and lysosomal rupture, activating the NLRP3 inflammasome (43). Further, cholesterol delivered by ox-LDL prevents cholesterol efflux in macrophages in a similar lysosomal-dependent manner (44) and causes the downregulation of ABCA1 at both the protein and mRNA levels in vascular endothelial cells (45). Alongside its detrimental effects on cholesterol transport, ox-LDL is directly implicated in atherosclerosis. Through binding to lectin-like oxidized low-density lipoprotein receptor (LOX-1), ox-LDL induces a variety of pro-atherogenic effects, such as foam cell formation as well as endothelial activation and apoptosis (46). Beyond oxidized HDL and LDL, other modified lipids like lipoprotein (a) (Lp(a)) can disrupt cholesterol efflux and increase inflammation. Lp(a) has a robust inverse association with CEC and directly disrupts plasminogen-dependent cholesterol efflux (47). Phospholipids within Lp(a) can be oxidized, further increasing its atherosclerotic effects, such as inducing arterial wall and macrophage-driven inflammation (48). These oxidized phospholipids can also carry and dispense MCP-1, further intensifying vascular inflammation (49).

## Human models of inflammation and dyslipidemia

Psoriasis is a chronic inflammatory, immune-mediated skin disease affecting 2-3% of the adult US population (50–52). Psoriasis patients are at high-risk of developing early-onset CAD, with an increased prevalence of non-calcified coronary plaques that are vulnerable to rupture, often leading to MI (3, 51, 53). In psoriasis, the prevalence of non-calcified coronary burden has a robust correlation with abnormal cholesterol trafficking, evident by impaired CEC, an elevation in circulating neutrophils that are activated, and increased activity in the bone marrow and spleen determined by heightened uptake of fluorodeoxyglucose (FDG) (2, 36, 54). The impairment in HDL function may partially be attributed to the elevation of oxidized LDL and oxidized HDL that are associated with the severity of coronary disease (55). *In vitro* studies of HDL function showed that macrophages loaded with oxidized LDL did not release cholesterol to HDL (44). Moreover, oxidized LDL accumulation has been reported in psoriatic lesions, most likely due to ineffective removal by HDL (56). Further, in psoriasis, the proteome of HDL various compared to healthy controls, leading to reduced CEC (57). HDL from patients with psoriasis had a lower concentration of ApoA-1 and ApoM. In exchange, the acute-phase proteins prothrombin, alpha-1-acid glycoprotein 1 and serum amyloid A were increased in HDL (57). Moreover, HDL particle size is smaller in psoriasis, which are known to efflux cholesterol less efficiently than their larger HDL counterparts and this finding is associated with aortic vascular inflammation by FDG-PET (20). Similar to murine models of atherosclerosis, psoriasis patients are inordinately inflamed, perpetuating abnormal cholesterol handling that further drives an overactive immune response (37). Currently, it is not known if cholesterol accumulation occurs in psoriasis bone marrow as a result of abnormal HDL function, however, ongoing evidence prompts future studies.

The evidence of interdependent pathways linking cholesterol transport and chronic inflammation extends beyond psoriasis. SLE has similar findings of altered lipid metabolism, with various lipid metabolites, such as arachidonic acid correlating with disease severity (58) and increased HDL oxidation, due to MPO released by neutrophils during NETosis (59). Due to the heightened state of oxidation impairing HDL function, patients with SLE have reduced CEC, which is associated with the increased cardiovascular disease found in SLE patients (60). Further, HDL derived from SLE patients has been shown to promote NF-kB signaling, suggesting SLE HDL can activate the inflammasome, promoting NETosis, further compromising un-modified HDL (61). Low-density granulocytes, a type of neutrophil increased in SLE and associated with atherosclerosis, has been shown to have an inverse relationship with CEC, suggesting heightened neutrophil driven impairment of cholesterol trafficking in the disease (62). T-cell activation in SLE is also heightened, in part due to impaired CEC. T-cell lipid raft formation and antigen expression is enabled by dysfunctional ApoA-1 and the inability of HDL to lower both cholesterol and major histocompatibility class II protein levels (63). Impaired CEC in SLE therefore could partially be responsible for increased T-cell activation in SLE (64). As a consequence of increased activation, the presence of ApoA-1 antibodies has been noted in SLE (65). Destruction of ApoA-1 further hampers efflux, and ApoA-1 antibodies have been shown to correlate with SLE disease activity.

Patients with rheumatoid arthritis (RA) also suffer from similar lipoprotein dysfunction. In RA, there is an increased presence of oxidized LDL, possibly due to increased neutrophilic production of ROS (66). Further, similar to psoriasis, CEC is negatively associated with disease severity in RA patients. Interestingly, while there is no significant difference in CEC between RA and controls, there is a stark decrease in CEC between those with major disease, when compared to those with little to no disease activity (67). Further, CEC in RA is negatively associated with MPO levels, comparable to SLE (67). Complementing this, plasma from RA patients was found to increase foam cell formation, downregulating cholesterol efflux proteins such as ABCA1, while upregulating scavenger receptors such as cluster of differentiation (CD)36. Finally, in a study of the synovial fluid lipidome of RA patients, 135 synovial lipids were found to be associated with disease activity and leukocytosis, suggesting a large amount of crosstalk between lipid meditators and inflammatory activity (68). Strikingly, these associations and lipidome changes were shown to occur before disease onset in the preclinical stage, indicating that these lipidome changes are not simply a result of RA induced inflammation (68). The literature shows regardless of the etiology of systemic inflammation, common consequences are HDL dysfunction, failures in cholesterol transport and overall lipid dysfunction.

## Inflammation as a therapeutic target

Abnormal cholesterol handling may contribute to the interdependence of inflammation and dyslipidemia and represents a critical area of research to elucidate therapeutic targets in cardiovascular pathologies. While treatment of cardiovascular diseases has benefited from the use of LDL-lowering drugs, residual inflammatory risk suggests efforts should be made to target both lipid dysfunction and inflammation. Over 40% of cardiovascular events occur in individuals with normal LDL levels (69), highlighting that isolated efforts to decrease LDL quantities do not remove all cardiovascular risk. This interdependent relationship was further supported in the JUPITER trial. Patients on statin therapy had a reduction in cardiovascular events only if both their LDL and inflammatory C-reactive protein (CRP) levels dropped (5). Finally, the Canakinumab Anti-Inflammatory Thrombosis Outcome Study (CANTOS) was pivotal in showing that targeting inflammasome driven inflammation with canakinumab, a human IgGκ monoclonal antibody targeting IL-1β that was developed by Novartis for the treatment of immune disorders, lowered cardiovascular risk (6). Systemic inflammatory conditions provide clinical models to understand how anti-inflammatory therapy may alleviate lipid dysfunction. In a recent study, HDL was isolated from 15 psoriatic patients (at baseline and after anti-psoriatic therapy) and from 15 age- and sex-matched healthy controls (70). Anti-psoriatic therapy was associated with improved serum lecithin-cholesterol acyltransferase activity (LCAT) and improved CEC suggesting that abnormal lipid trafficking in chronic inflammatory conditions is improved upon treatment of inflammation (70). In SLE, a randomized, double-blind, placebo-controlled clinical trial of tofacitinib associated with improved HDL cholesterol levels and particle number, as well as improved CEC (71). Finally, treatment of rheumatoid arthritis reduced inflammation as measured by high sensitivity C-reactive protein, which associated with improved CEC (72). These findings illustrate the potential of solving the challenge of lipid trafficking through the treatment of inflammation and represent a paradigm shift towards a focus on treating inflammation and dysfunctional lipoprotein metabolism as connected entities.

## Cholesterol trafficking as a therapeutic target

New treatment efforts to combat dyslipidemia include focusing on cholesterol metabolism and trafficking. Much like treatment of inflammation is associated with improved lipid trafficking, mimetics have also shown to be independently associated with ameliorated lipoprotein metabolism. ApoE mimetic peptide therapy, for example, may be ideally suited for the treatment of hypercholesterolemia and may provide additional benefits when compared with alternatives such as PCSK9 inhibitors (73). Two ApoE mimetic peptides - Ac-hE18A-NH (2) and mR18L – had a similar reduction in plasma cholesterol and atherosclerotic lesion areas (74). Moreover, ApoA-I mimetic peptides are potential therapeutic agents for increasing CEC (75). More recently, however, there has been interest in the potential of recombinant LCAT in improving cardiovascular disease and abnormal lipoprotein profiles. In thirty ST-segment-elevation myocardial infarction patients, *in vitro* incubation of plasma with recombinant human LCAT restored the ability of HDL to promote endothelial nitric oxide production, suggesting that LCAT is a potential therapeutic target for restoring HDL function (76). Moreover, infusion of A12 antibodies into mice lowered plaque formation and reduced circulating free LDL (76). These findings suggest that anti-ALDH4A1 antibodies and recombinant LCAT improves lipid trafficking and may have therapeutic efficacy in cardiovascular disease. Indeed, interest in this area has commenced clinical trials to investigate further the potential of such therapeutics, such as REAL-TIMI 63B, a phase 2b randomized study to evaluate the efficacy, safety, pharmacodynamics, and immunogenicity of repeat doses of MEDI6012 in adult subjects presenting with acute STEMI. Another field of growing interest, is the use of genetic studies to elucidate associations between rare gene variants and dyslipidemia, thus providing pharmacological targets. For example loss-of-function variants in the ANGPTL3, a lipoprotein lipase inhibitor, leads to lower plasma levels of LDL-cholesterol and triglycerides (77), concomitant with reductions in coronary artery disease (67). Therefore, ANGPTL3 is currently a pharmacological target for the treatment of cardiovascular disease (CVD) (78). Experimental evidence demonstrates that anti-ANGPTL3 therapies have a significant lipid lowering effect from phase I clinical trials with an anti-ANGPTL3 antibody (evinacumab) (79) and anti-sense oligonucleotide targeting ANGPTL3 (80). However, it remains undetermined if this lipid lowering therapy decreases cardiovascular events.

## Conclusion

Future efforts should be directed to further understand the interplay between inflammation and cholesterol handling and expand upon the nascent clinical studies implicating dysfunctional cholesterol trafficking as a cause of immune cell expansion. Treatment strategies for cardiovascular disease and systemic inflammatory conditions should begin to treat dyslipidemia and inflammation as connected entities rather than two distinct pathologies. Further work is needed to parse out the role CEC plays in humans to help mediate these complex relationships.

## Author contributions

RO’H, NM and HT were involved in designing the concept of the review and oversight. RO’H, AB and HT were involved in the literature search, summation of the literature, and revisions of the manuscript. RO’H, AB, CH, PP, and HT drafted the manuscript. All authors contributed to the article and approved the submitted version.

## Funding

Supported by the National Heart, Lung, and Blood Institute Intramural Research Program (HL006193-07). This research was made possible through the NIH Medical Research Scholars Program; a public-private partnership supported jointly by the NIH and contributions to the Foundation for the NIH from the Doris Duke Charitable Foundation, Genentech, the American Association for Dental Research, the Colgate-Palmolive Company, and other private donors.

## Conflict of interest

NM is a full-time US government employee and has served as a consultant for Amgen, Eli Lilly, and Leo Pharma, receiving grants/other payments; as a principal investigator and/or investigator for AbbVie, Celgene, Janssen Pharmaceuticals, Inc, Novartis, and AstraZeneca receiving grants and/or research funding; and as a principal investigator for the National Institute of Health receiving grants and/or research funding.

The remaining authors declare that the research was conducted in the absence of any commercial or financial relationships that could be construed as a potential conflict of interest.

## Publisher’s note

All claims expressed in this article are solely those of the authors and do not necessarily represent those of their affiliated organizations, or those of the publisher, the editors and the reviewers. Any product that may be evaluated in this article, or claim that may be made by its manufacturer, is not guaranteed or endorsed by the publisher.
